# Postural Adaptation of the Spatial Reference Frames to Microgravity: Back to the Egocentric Reference Frame

**DOI:** 10.1371/journal.pone.0010259

**Published:** 2010-04-20

**Authors:** Sébastien Viel, Marianne Vaugoyeau, Christine Assaiante

**Affiliations:** Groupe DPA, Pôle 3C - UMR 6149, Université de Provence & CNRS, Marseille, France; Mount Sinai School of Medicine, United States of America

## Abstract

**Background:**

In order to test how gravitational information would affect the choice of stable reference frame used to control posture and voluntary movement, we have analysed the forearm stabilisation during sit to stand movement under microgravity condition obtained during parabolic flights. In this study, we hypothesised that in response to the transient loss of graviceptive information, the postural adaptation might involve the use of several strategies of segmental stabilisation, depending on the subject's perceptual typology (dependence - independence with respect to the visual field). More precisely, we expected a continuum of postural strategies across subjects with 1) at one extreme the maintaining of an egocentric reference frame and 2) at the other the re-activation of childhood strategies consisting in adopting an egocentric reference frame.

**Methodology/Principal Findings:**

To check this point, a forearm stabilisation task combined with a sit to stand movement was performed with eyes closed by 11 subjects during parabolic flight campaigns. Kinematic data were collected during 1-g and 0-g periods. The postural adaptation to microgravity's constraint may be described as a continuum of strategies ranging from the use of an exo- to an egocentric reference frame for segmental stabilisation. At one extremity, the subjects used systematically an exocentric frame to control each of their body segments independently, as under normogravity conditions. At the other, the segmental stabilisation strategies consist in systematically adopting an egocentric reference frame to control their forearm's stabilisation. A strong correlation between the mode of segmental stabilisation used and the perceptual typology (dependence - independence with respect to the visual field) of the subjects was reported.

**Conclusion:**

The results of this study show different subjects' typologies from those that use the forearm orientation in a mainly exocentric reference frame to those that use the forearm orientation in a mainly egocentric reference frame.

## Introduction

Posture control is integral to the execution of goal-directed action and underlies the ability to control movement under various contexts and environments. An important function of posture is to ensure maintenance of balance during the initiation, continuance and completion of action. In addition, posture serves as a reference frame for the production of accurate movements. Indeed, the efficient action of the body in space and its representation need that the central nervous system (CNS) uses a reference frame around which the external objects' positions and displacements could be estimated and movements can be built up. A question asked frequently in motor neuroscience surrounds the problem of the existence of a stable reference frame used to control posture and voluntary movement [1], [2]. On Earth, there exist two main postural reference frames: 1. the exocentric reference frame, which is based mainly on the gravity vector and on visual cues and 2. the egocentric reference frame, which is based on either the subject's whole body or on the segments engaged in an ongoing action. In contrast, under microgravity condition, the exocentric reference frame is perceived only on the basis of the visual cues available in the subject's immediate environment, i.e. those provided by the spacecraft or aircraft cabin; whereas the sensory messages mediating ongoing actions in the egocentric frame are largely affected under these conditions because they are associated with a decrease in proprioceptive inputs [3].

Microgravity has always provided privileged conditions for studying the body scheme. The body scheme involves an overall representation, including parts of both the exocentric and egocentric reference frames. Indeed, the postural body scheme is normally used to control posture according to a feed-forward process, based on internal representations such as the body geometry, the forces exerted on the ground and the orientation of the subject's body in relation to the vertical pull of gravity [4], [5].

Under microgravity conditions, if the visual cues are abolished, the only sensorial information which can be used by subjects to orient their bodies in relation to the spacecraft results from the integration of the haptic information with the proprioception of all joint of the kinematic chain involved in the movement. This information participates to the recalibration of the body scheme [6]. Haptic cues can originate from the hands, for instance, when subjects are leaning on their hands or from the instep and ankle joint when subjects' feet are attached to the floor of the cabin. In the latter case, subjects can use plantar information about the orientation of the floor to adjust the angular positions of the whole chain of axial joints suitably in order to adopt a standing posture [7], or to adjust the orientation of any of the body segments, using the ascending system described by Mergner and Rosemeier [8] in their conceptual model. Lackner and Graybiel [9] have shown, however, that the sense of relative body configuration is preserved under microgravity based on body scheme.

Exposure to microgravity also provides a privileged situation for studying the control of spatial orientation. It has been proposed that an internal representation of body vertical has a prominent role in spatial orientation. Recent study during parabolic flight [6] investigating the ability of human subjects to accurately locate their longitudinal body axis while free-floating in microgravity, reported that mechanical pressure on the chest improved spatial orientation. Indeed, microgravity selectively abolishes the somaesthetic graviceptive information and the static otolithic information. Lastly, vision plays a particularly important role under weightlessness, since it contributes in particular to recalibrating other sensory components such as those mediated by the proprioceptive system, which is affected under weightless conditions [10]. The subjects' reliance on the visual reference frame immediately increases in space, whereas their reliance on graviceptive and proprioceptive cues has been found to decrease during parabolic flight [11] as well as during spaceflight [12]. This increased reliance on visual cues may be accompanied under weightless conditions by a change in the postural orientation and stabilisation strategies adopted. The great reliance on visual cues observed in subjects exposed to experimental microgravity condition could be directly due to the lack, or impairment, of the other sources of information i.e otholitic and somaesthetic graviception.

A similar importance of the role played by visual cues, when the other sensorial sources are not reliable, has been shown also in many developmental studies from infancy up to the age of 6 years [13] and during some of the key stages in ontogenesis, such as adolescence [14] [15] and in neurological disorders such as Parkinson's disease [16], deafferented patient [17] vestibular subjects and visual vertigo patients [18].

In the present study, we adopted the working hypothesis that, because of the lack of graviceptive information, the postural adaptation of the spatial reference frames to microgravity might involve the use of exocentric reference. This hypothesis was tested by applying during parabolic flights a forearm stabilisation task during trunk movement. This forearm stabilisation task combined with a sit to stand (STS) movement was adopted to study how segmental orientation and segmental stabilisation of both forearm and trunk are controlled under microgravity conditions, with a view to showing, in adults, a back to the egocentric reference frame. We have shown in previous study [19] that under microgravity condition the kinematics characteristics of STS movement were modified as compared with the same movement made under terrestrial condition. More precisely, the amplitude of the trunk bending was drastically decreased under microgravity condition. Because in the present study, the STS movement was used only in order to perturb trunk orientation, we have standardized STS movement by asking to the subject to bend the trunk to an angle of 45°.

Lastly, we hypothesize that the postural performances of our subjects in term of segmental stabilisation would be correlated with the subjects' perceptual performances, as it is has been previously been reported on earth in healthy adults [20], [21].

## Materials and Methods

### 1. Subjects

11 healthy subjects (8 males and 3 females) participated in this study. They gave their informed consent prior to the experiment, which obtained the approval of the local ethics committee and have therefore been performed in accordance with the ethical standards of the Declaration of Helsinki. Subjects had passed medical tests to qualify for the parabolic flights (i.e., the equivalent of an Air Force Class III medical examination). All the participants had previous experience in parabolic flights. Nine of the subjects were given ScopDex, a drug which alleviates motion sickness. In order to evaluate eventual differences between subjects with and without ScopDex, a Mann-Whitney Rank Sum Test was realised on the postural parameters. Because no difference between subjects with and without ScopDex was found for either normal or microgravity conditions, we did not make a separate analysis.

### 2. Experimental procedure

Experiments were performed during two parabolic flight campaigns (3 flights per campaign) on the French Airbus A300 aircraft. Each flight lasted for about 2h30, and included 30 parabolic free-fall episodes each lasting for about 22 seconds in the Airbus aircraft, and resulting in a gravity level of about 0.02g. All the parabolas recorded had the same pattern: the pull-up phase usually stabilised at a gravity level of around 1.75g for 20-22 seconds and was followed after a 4-second interval by a 0-g phase. The pull-out phase also consisted of a 1.75-g period lasting for 15 seconds, followed by the return to the 1-g level. The intervals between two successive parabolas lasted for 2 minutes.

As it was reported in many previous studies [22], [23], [24], [25], [26], trials were run alternately under microgravity and normogravity conditions, and the same paradigm was used under both gravity conditions. Given the shortness of the microgravity episodes, only one trial could be performed during each parabolic phase.

### 3. Experimental task

The subject had to adopt an upright posture after performing a sitting to standing movement. Before each trial, the subject was seated on a chair with both feet fixed to the ground, the knees bent at an angle of 90°, the head and trunk held straight, the right hand holding onto the chair and the left arm placed horizontally. Two seconds after the beginning of the kinematic recording, the subject had to bend the trunk to an angle of 45° in response to a vocal signal and stand up as quickly as possible, still keeping the left forearm in the horizontal position. In order to accentuate the stabilisation's instruction, the subject had to wear a glove containing a 1-kg weight on the left hand. The subject was asked to maintain the vertical standing position with their arm extended horizontally until instructed to relax. This task was run only with eyes closed, under 1-g and 0-g conditions.

### 4. Data acquisition

**Figure 1:**
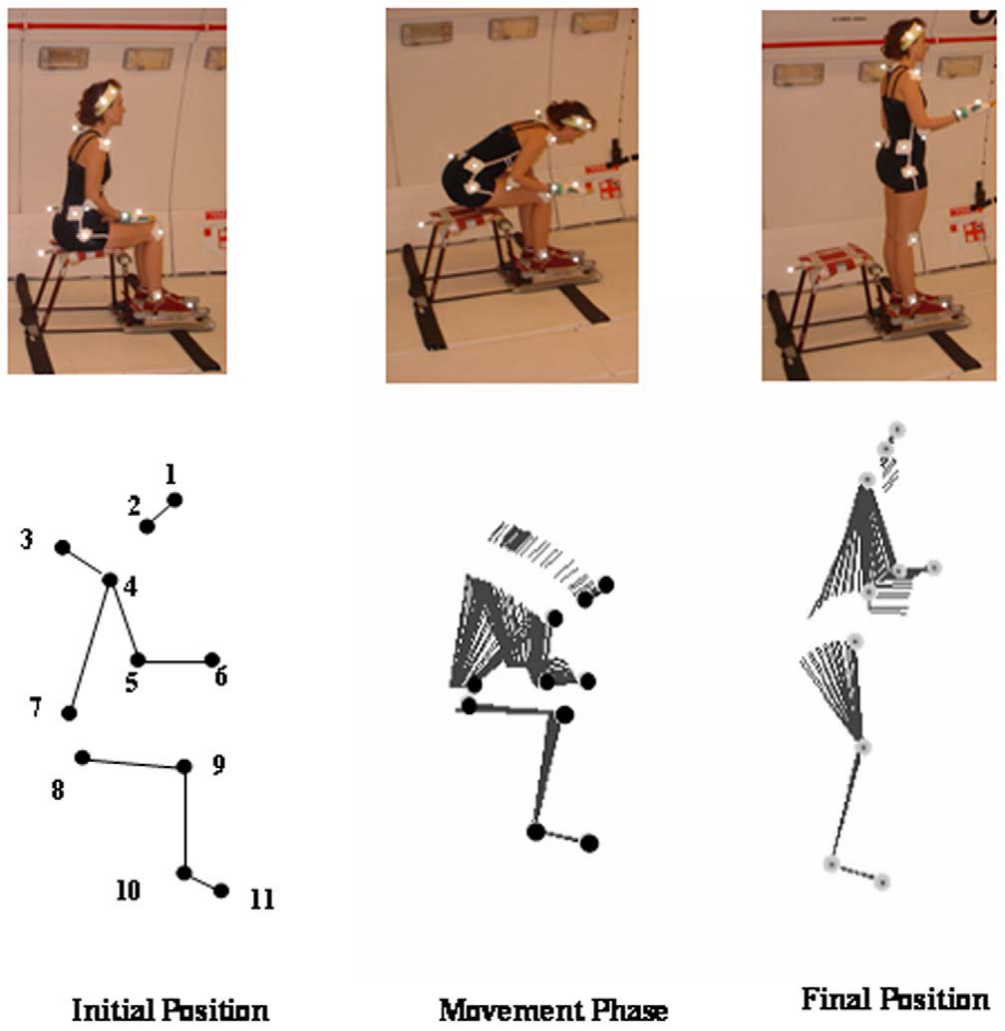
Experimental design. Upper part: Photography of one subject making the task in 1 g. part a corresponds to the initial position, part b to the movement phase and part c to the final position. Lower part: Left side: Arrangement of the 11 markers used to analyse the forearm stabilisation during a sit to stand task. The 11 markers were placed at the following sites: (1) at the top of the head, (2) at the level of the external angle of the eye orbit, (3) on C7, (4) on the left shoulder acromion, (5) on the left elbow, (6) on the left wrist, (7) on the left antero-superior iliac spine, (8) on the left great trochanter, (9) on the left knee, (10) on the left external malleolus and (11) on the 5^th^ metatarsien. Right side: Stick diagram of the sit to stand movement,

Data were collected during both 1-g and 0-g periods using an optoelectronic system (SMART eMotion). 4 infrared-emitting cameras (sampling rate 120 Hz) recorded the movements of 11 retroreflective markers (diameter 10 mm). These markers were placed from top to the bottom as follows: at the top of the head, at the level of the external angle of the eye orbit, on C7, on the left shoulder acromion, on the left elbow, on the left wrist, on the left antero-superior iliac spine, the left great trochanter, the left knee and the left internal malleolus and on the 5^th^ metatarsien (figure 1).

The field of view explored was 2×2×3.5 m and the accuracy was thus to within about 1 mm.

### 5. Determination of movement phases

3 movement phases were determined on the basis of the antero-posterior movements of the marker placed on C7. The first phase corresponded to the subject's starting position. The second phase corresponded to a trunk bending movement in the antero-posterior plane following by the sitting to standing movement. The third phase corresponded to the final position, where the subject had to maintain a final upright standing posture, keeping the left arm raised horizontally. Here, we mainly focus our analysis on the second phase.

### 6. Data analysis

The absolute angles (with respect to the external axis) around the pitch axes of the trunk, arm and forearm were computed every 8.3 ms during each trial. On the basis of these angular values, the values of several controlled variables were calculated to obtain segmental angular dispersion and stabilisation values (anchoring index).

#### a. Segmental Angular dispersion

At each trial, the standard deviation (the dispersion, denoted *σ* (*θ*_a_)) of the absolute angular distributions of the forearm was calculated in the sagittal plan during the trunk inclination. Thus, the angular dispersion gives an indication of the amplitude of the forearm movement in the sagittal plane. A little angular dispersion indicates little forearm movement, whereas, a great angular dispersion indicates great forearm movement.

#### b. Anchoring Index

Segmental stabilisation of the forearm was defined in terms of the anchoring index calculated during the performance of the task [27], [28], [29], [30], [31].

The anchoring index was used to determine the stabilisation of the forearm with respect to both space and the trunk.

With regard to the forearm AI for example, the angular orientation of the forearm relative to the trunk was first calculated every 8.33 ms during a trial using the formula:
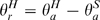



In this formula, 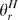 is the angular orientation of the forearm relative to the trunk, and 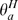 and 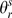 are the absolute forearm and trunk angular orientations, respectively.

For each trial, the standard deviation of the relative angular distribution (σ_r_) and the standard deviation of the absolute angular distribution (σ_a_) were calculated for the forearm.

**Figure 2:**
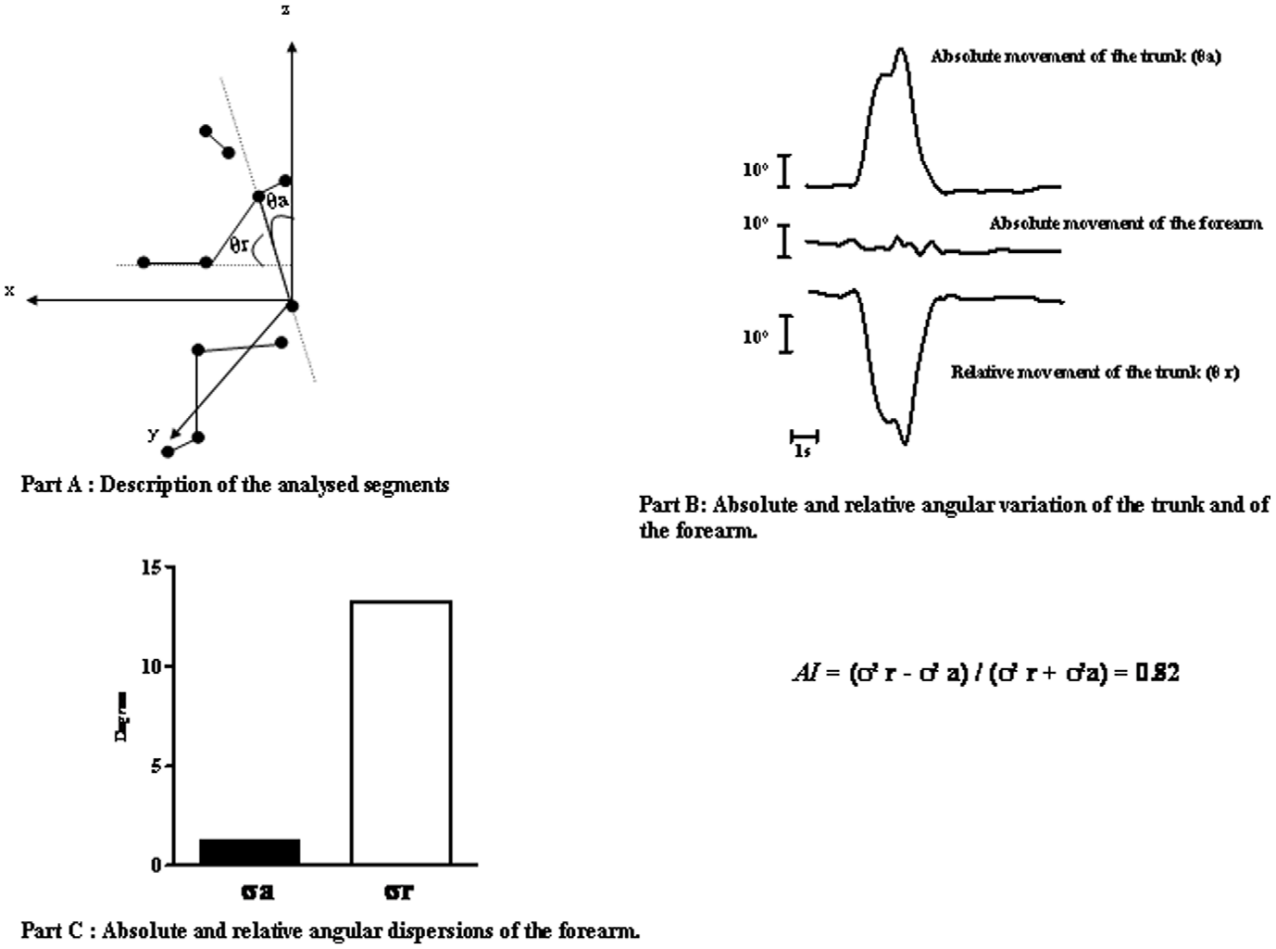
Anchoring Index calculation. Part A: Diagram of the trunk pitch angle with respect to the external axis, θ_a_, and the forearm pitch angle with respect to the trunk, θ_r_. With x: lateral axis, y sagittal axis and z vertical axis. Part B: angular pitch displacement of the trunk (upper trace), the forearm (middle trace) and the relative angular movement of the forearm with respect to the trunk (lower trace). Part C: Diagram of the absolute (σ_a_) and relative (σ_r_) pitch dispersions of the forearm, according to the definition of the anchoring index (AI). In this example, AI is positive, which means that the forearm is stabilised in space independently of the trunk movements.

The segmental anchoring index was used to compare the stabilisation of a given segment (in this study the forearm) with both an external reference value and the value obtained on another body segment (in this study the trunk) (part B of figure 2). This index was calculated at each trial as follows, as shown in figure 2.
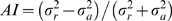
where σ_a_ is the standard deviation of the angular distribution about the pitch axis of the segment under investigation with respect to the external reference value (the absolute vertical direction defined during the calibration of the system before flight. In this condition the vertical external reference was parallel with the vertical lines of the airplane, and the horizontal axis were parallel with the plane's floor) and σ_r_ is the corresponding standard deviation of the angular distribution with respect to the underlying segment (part A and C of figure 2). A positive value indicates that a better segmental stabilisation has occurred on the absolute vertical or horizontal axis than on the underlying segment, whereas a negative value indicates that better segmental stabilisation has occurred on the underlying segment. The anchoring indexes of the forearm with respect to the trunk were calculated during each movement phase.

### 7. Rod and Frame test (RFT)

In order to determine the dependence–independence with respect to visual field of the subjects, subjects were tested on the RFT apparatus [32], on earth before flight. The subject had to estimate the subjective vertical (SV) by means of a little bar placed in a square frame, which could be tilted to either the right or the left (18°). The frame tilt of ±18° was chosen, because with this inclination the frame effect has been found to be maximal in previous studies [33], [34], [35].

Under these conditions, the frame effect which reveals the error in the vertical subjective due to the tilted frame was calculated according to the method of Nyborg et Isaksen [36]. The subjects under investigation are usually simply divided into visually independent subjects (those making errors below the median value) and visually dependent subjects (those making errors above the median value).

### 8. Statistical analysis

15 trials were run with each subject on each of the data analysed under both gravity conditions. Descriptive statistics are given in the form of medians and interquartiles.

Anchoring indexes were compared to zero, using a single-sample analysis (t-test) against the null hypothesis. Since these indices were in the −1 to +1 range, we used a z transform to convert the values into an unbiased Gaussian distribution. The effects of gravity conditions on postural orientation and stabilisation performances were tested using Wilcoxon's signed rank test for within-subject comparisons. Differences between Independent and dependant subjects were tested with a Mann-Whitney U test. The relationship between the perceptual category to which the subjects belonged and the segmental stabilisation strategy used was assessed in terms of the Spearman coefficient of correlation between the forearm anchoring index and the corresponding error made by the subjects in the RFT. Differences with a p value <0.05 were considered to be statistically significant.

## Results

### 1. Overall analysis

#### a. Checking that the instructions were carried out

In order to check that the instructions about bending the trunk before getting up from the chair had been properly carried out; the maximum trunk bending angle was measured in the antero-posterior plane. Under both gravity conditions, the subjects were found to have obeyed the instructions on the whole, since the mean trunk bending angle was found to be 42° (+/−7.3°) under normogravity conditions and 46° (+/−5°) under microgravity conditions.

#### b. Forearm dispersions and anchoring indices

**Figure 3:**
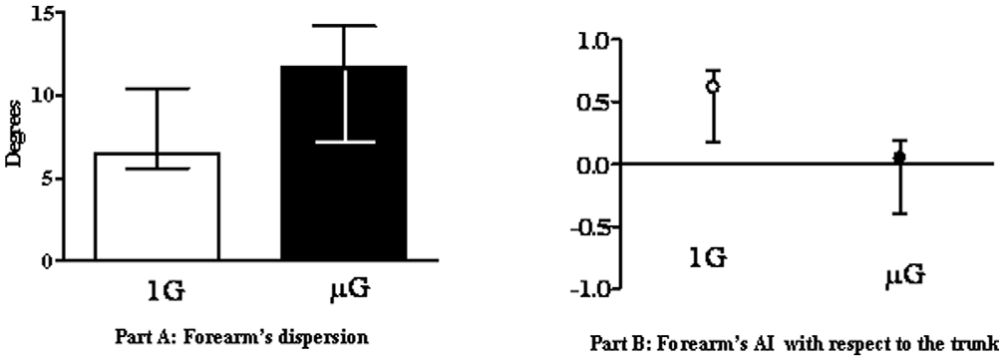
Angular dispersions : Part A: Medians and 1st and 3rd quartiles of dispersions of the absolute forearm's angle in normogravity (white) and microgravity (black). Part B: Medians and 1st and 3rd quartiles of forearm anchoring indexes (AI) under normogravity (white) and microgravity (black).

The medians and quartiles of all the subjects' forearm angular dispersions (part A of figure 3) and the forearm anchoring indices calculated in relation to the trunk under both gravity conditions (part B of figure 3) are given in figure 3.

Statistical analysis of the forearm angular dispersions (part A of figure 3) showed the existence of a significant difference between the two gravity conditions (W = 66, p<0.001). The forearm angular dispersion was greater under microgravity than under normogravity conditions (13° and 6°, respectively).

Comparisons between the forearm anchoring indices recorded between the two gravity conditions (part B of figure 3) showed the existence of a significant difference (W = 66 p<0.01). The values of the anchoring indices obtained under normogravity conditions were significantly positive (t = 3.898; p<0.01), which suggests that the forearm was efficiently stabilised in space, despite the perturbing antero-posterior trunk movements involved in the performance of the task. These results suggest that the forearm and trunk were controlled independently. Under microgravity conditions, on the contrary, the anchoring indices did not differ significantly from 0.

### 2. Individual analysis

Under microgravity we have shown a global decrease of the AI values. With a functional point of view, this decrease may reflect at least 2 kinds of adaptive strategies:

1. The AI values decrease but remain positive: This indicates a decrease of the forearm's stabilisation with respect to space
2. The AI values shifted from a positive to a negative value: This underlines that the strategy of forearm's stabilisation with respect to space was transitory loosen.

**Figure 4:**
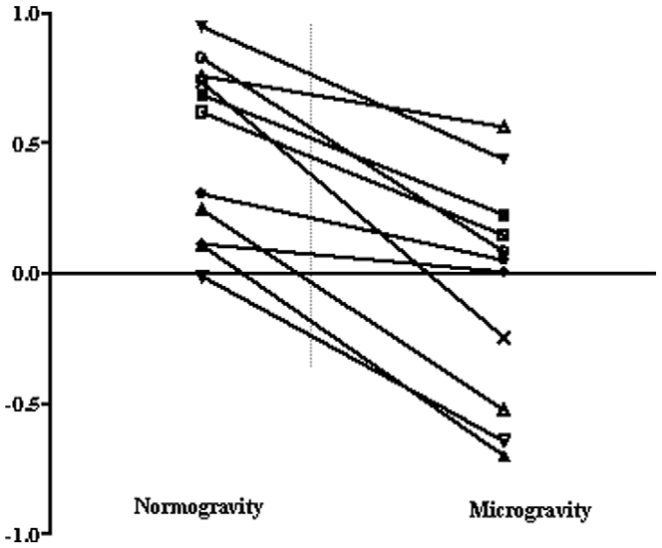
Individual analysis. Mean anchoring indexes of the forearm of each subject under normogravity (left) and microgravity (right) conditions. Each point represents the averaged values for one subject in the given experimental condition.

With these results in mind it appears to be relevant to present each subject performances. Figure 4 gives the values of the anchoring indices obtained with each subject under normogravity and microgravity conditions.

Under microgravity condition, we showed that the subjects' strategies followed a continuum between two opposite strategies: 1) a strategy whereby the forearm was couple to the trunk (IA<O), and 2) a strategy whereby the forearm and trunk were controlled separately (IA>0).

### 3. Relationship between segmental stabilisation strategies and perceptual typologies

**Figure 5:**
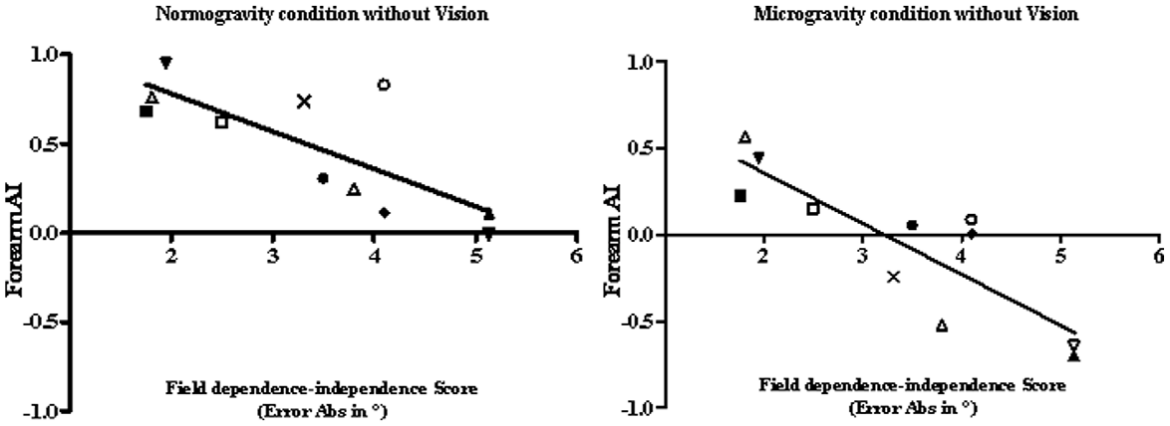
Relation between postural and perceptive performances. Coefficient of correlation between field dependence–independence score and the corresponding AI of the forearm under microgravity condition. Each point represents the averaged values for one subject in the given experimental condition.

Whether the perceptual typology of the subjects may have affected the subjects' postural performances was tested by calculating a Spearman coefficient of correlation between the forearm anchoring index values with the adjustments made in the RFT of each subject under normogravity and microgravity conditions. The results obtained are given in figure 5.

The Spearman, coefficient of correlation shows that the subjects' postural performances (i.e the anchoring indexes) were significantly correlated with their perceptual performances (i.e the adjustment errors made in the RFT) under normogravity (r = −0.68; p<0.001) as well as in microgravity (r = −0.85; p<0.001). In both gravity condition, the lowest anchoring index values were obtained by the subjects making the highest adjustment errors, whereas the highest anchoring index values were obtained by those making the lowest adjustment errors, and there was a continuum between the two extremes.

In other words, under normogravity condition as well as under microgravity condition the visual independent subjects (VI) performed a better forearm stabilization on space than visual dependent subjects as indicated by their greater AI values. More specifically, under normogravity condition the perceptual typology influences the strength of the segmental stabilisation but not the choice of the reference frame used for stabilising as indicated by the positive values of AI whatever the subjects' typology. Under microgravity condition, the choice of reference frame seems to depend on the perceptual typology. The strategy whereby the forearm was stabilised with respect to space was mainly adopted by subjects who were fairly insensitive to visual perturbations, i.e. by VI subjects, and the strategy whereby the forearm was stabilised with respect to the trunk was mainly adopted by VD subjects, i.e. those who were dependent upon the visual field.

## Discussion

Under normogravity conditions, the positive anchoring index values suggest that the subjects maintained their forearm horizontal despite the perturbations caused by shifting from a sitting to standing position. Under microgravity condition, with eyes closed, most of the sources of information which could be used to build an exocentric representation of the movement are not available (vision and graviceptive information). It is worth noting, that in these conditions, the subjects did not all use the same adaptive postural strategy in response to microgravity.

### Continuum of postural strategies

Our results show a continuum of anchoring index values' decay, from positive to negative values. This underlines the existence of a continuum of segmental stabilisation strategies. More precisely, the forearm AI used in order to determine the referential frame used to stabilize the forearm during task decreased drastically under microgravity condition for all subjects. With a functional point of view, under microgravity condition, in some subjects the AI values remained positive, just indicating a decrease of the forearm's stabilisation with respect to space whereas in the other subjects the AI values shifted from a positive to a negative value indicating that the strategy of forearm's stabilisation with respect to space was loosen. In other word, some subjects maintained forearm's stabilisation with respect to space in order to keep it in practically the same position during the performance of the task, whereas the others adopted a strategy consisting in stabilising the forearm with respect to the trunk by coupling the trunk strongly to the forearm. Therefore, these subjects had greater difficulty in keeping their forearm horizontal while shifting from the sitting position to upright stance via a forward leaning movement.

This variability in segmental strategies of stabilisation across subjects could be explained by differences in how individual subjects re-distribute the relative weighting between available sensory information under microgravity. Kluzik and collaborators [37] have reported similar variability among subjects concerning the trunk orientation control on an inclined surface on earth. Their study supports the idea that individuals vary in the degree to which they weight proprioceptive, kinematic versus vestibular, graviceptive information for their postural orientation. Isableu and Vuillerme [38] have reported that the exploitation of the kinaesthetic relationships to postural control varied from one subject to another. This explanation can also be put forward in order to explain the different strategies across subjects of our study. Indeed, under microgravity conditions, if the visual and graviceptive cues are abolished, the only sensorial information which can be used by subjects to orient their bodies in relation to the spacecraft results from the integration of the haptic information with the proprioception of all joint of the kinematic chain involved in the movement. Subjects can use plantar information about the orientation of the floor to adjust the angular positions of the whole chain of axial joints suitably in order to adopt a standing posture [39], and to adjust the forearm orientation, using the ascending system described by Mergner and Rosemeier [40] in their conceptual model.

In this case, the difficulty to stabilize the forearm on the horizontal axis in some subjects could derived from a difficulty to process the proprioceptive cues, via the ankle, of the body orientation relative to the floor which still exist and persist in microgravity, as long as the feet are attached to the floor. The other subjects, favouring a proprioceptive body/floor angular coding, via the ankle, presented under microgravity no difficulty in maintaining horizontal forearm's orientation.

### Keeping an exocentric reference frame

Despite the absence of visual cues and of graviceptive information, some subjects were able to maintain a horizontal forearm's position. This indicates that they were able to build an external reference frame by integrating angular displacement of all joints involved in STS movement, and/or dynamics proprioceptive cues stemming from the inertia moment, of our limbs with the haptic (cutaneous plantar cues) and vestibular (coming from head acceleration) information in order to estimate the absolute angle of the forearm (body scheme) and maintain it in horizontal position. This suggests also that this ability to independently control the forearm and trunk under microgravity condition might be based on the use of the postural body scheme [10]. Other authors have used the body scheme concept to explain subjects' ability to stabilise their head in space despite the perturbations resulting from the voluntary trunk oscillations occurring under short-term and long-term microgravity conditions [41], [42]. Mergner and Rosemeier [43] have suggested that under microgravity conditions, simply making contact with the space cabin may suffice to update the interactions between the various sensory inputs involved in postural control. On the other hand, Lackner and DiZio [44] have established that in the absence of graviceptor activation, foot plant cues activate an internal model mediating perception of the Z-axis. Indeed, it has been proposed that an internal representation of body vertical has a prominent role in spatial orientation. A recent study during free-floating with no contact cues, reports that the normal longitudinal body axis perception is altered and that the normal accuracy is reinstated by the application of tactile cues on the subject's chest [6]. Nevertheless, in the present study, the subjects were able to use cutaneous plantar cues and the proprioceptive information of all joints of the kinematic chain involved in the movement execution not only to perceive the Z axis, but also to control the geometrical relationships between their various body segments.

### Back to the egocentric reference frame

By contrast, some of our subjects were unable to precisely perform the task, which is the horizontal stabilization of the forearm. This indicates that they probably were unable to integrate correctly haptic and vestibular information to control posture under microgravity. Their strategy, consisting in stabilising the forearm with respect to the trunk, reflects that they only used joint angle information to control forearm stabilisation. In this case, the trunk orientation seems to be used as the main reference frame for controlling the forearm's posture. This process, involving that the control of the horizontal forearm's position is based mainly on the use of an egocentric reference frame, have been previously described as occurring on Earth in young children aged 3–6 years [14], [45], [46]. The authors of a previous study [47] also suggested that it may have been by reverting to the use of an egocentric reference frame that two astronauts after spending 4 months under microgravity were able to recover their terrestrial posture/movement coordination performances in a forward trunk bending task despite the sensorimotor processing changes they had undergone. In addition, Friederici and Levelt [48] have also demonstrated that in weightlessness, subjects tended to localize object position in space by means of an egocentric reference. Consequently, movements could be estimated within a body-related reference situated at the head-trunk level. Pozzo et al. [49] reported that the strategy which consists of stabilising the trunk during rotation provides an egocentric reference frame used to calculate target position in extra corporal space, as well as necessary limb trajectory to reach the target.

### Correlations between perceptual strategy and choice of reference frame

The differences of the segmental strategy used among subjects could be attributed to the different subjects' expertise of microgravity. Nevertheless, all subjects had experiment parabolic flights before and it is important to note that this experience did not eliminate perceptivo-motor reliance on different frames of reference. Indeed, the results of this study show the existence of a strong correlation between the mode of segmental stabilisation used and the perceptual typology of the subjects.

Visually dependent (VD) subjects tended to use an egocentric reference frame to stabilise their forearm, whereas visually independent (VI) subjects tended to use an exocentric mode of control. The hypothesis put forward by Isableu et al. [50] that VD subjects might actually show proprioceptive neglect, whereas VI subjects might on the contrary rely mainly on proprioceptive cues may explain the differences observed between these two groups. In fact, despite the absence of gravity, the present VI subjects may have recalibrated their sensory integration processes, especially those involving plantar and ankle angle [51] which would enable them to form a representation of the horizontal position of the aircraft cabin and the geometry of the various body segments with respect to the cabin and to each other, and thus to keep their forearm in the horizontal position. By contrast, during this short period of adaptation, the VD subjects may have had difficulty in recalibrating the sensory processes required to update the internal exocentric control scheme, as described by Mergner and Rosemeier [52]. The short term adaptation occurring in present VD subjects therefore consisted in strongly coupling trunk and forearm and reactivating the exocentric control system based mainly on proprioceptive trunk signals generated by the ongoing movements.

*In conclusion*, the results of this study show different subjects' typologies from those which use the forearm orientation in a mainly exocentric reference frame to those which use the forearm orientation in a mainly egocentric reference frame. The first one use the same stabilising strategy as under terrestrial conditions to control each of their body segments independently, probably based on the body scheme and short term sensorimotor memory. In response to the sudden perturbations associated with the microgravity conditions occurring during parabolic flight, the others adopted an egocentric reference frame to control the position of their body. In these subjects, the trunk thus becomes the main reference frame to control the forearm stabilization and orientation under weightlessness.

Having thus established the existence of a strong correlation between the perceptual approach used by the subjects and their choice of reference frame, further experiments will assess the adaptive postural processes occurring under microgravity conditions in visually independent and dependent subjects performing a postural task in which they are required to adopt the upright position in a situation giving rise to a visuo-somesthetic conflict.
